# Histological transition from minimal change disease to THSD7A-associated membranous nephropathy in a patient receiving long-term steroid treatment: A case report

**DOI:** 10.1097/MD.0000000000035470

**Published:** 2023-10-13

**Authors:** Aki Kojima, Takahiro Uchida, Kentaro Sugisaki, Minami Koizumi, Ken Aoki, Mitsuya Mukae, Muneharu Yamada, Takashi Oda

**Affiliations:** a Department of Nephrology and Blood Purification, Kidney Disease Center, Tokyo Medical University Hachioji Medical Center, Hachioji, Tokyo, Japan.

**Keywords:** membranous nephropathy, minimal change disease, nephrotic syndrome, thrombospondin-type-1-domain-containing-7A

## Abstract

**Rationale:**

A predominant Th2 immune response is suggested in the pathogenesis of both minimal change disease (MCD) and membranous nephropathy (MN); however, consecutive development of the 2 diseases in a patient is extremely rare.

**Patient concern:**

A Japanese man, who developed nephrotic syndrome in his 50s and was diagnosed with MCD by renal biopsy, experienced a relapse of proteinuria approximately 3 years later during long-term steroid treatment. Since the proteinuria was resistant to increase in steroid dosage, repeat renal biopsy was performed, which revealed a small amount of glomerular subepithelial immune deposits containing immunoglobulin (Ig)G (dominantly IgG4). Immunostaining for thrombospondin-type-1-domain-containing-7A (THSD7A) was positive on the glomerular capillary walls, whereas that for other causative antigens of MN, such as phospholipase A2 receptor or neural epidermal growth factor-like 1 protein, was negative. Detailed examination found no associated condition, including malignancies and allergic diseases.

**Diagnosis:**

The diagnosis of THSD7A-associated idiopathic MN was made.

**Interventions and outcomes:**

He received further increased dose of steroids. Thereafter he maintained clinical improvement because his urinary protein level was decreased.

**Lessons:**

The present case suggested that histological transition from MCD to MN is possible and repeat biopsy would be crucial for accurate diagnosis.

## 1. Introduction

Minimal change disease (MCD) and membranous nephropathy (MN) are the leading causes of nephrotic syndrome in adults. MN is usually classified into the idiopathic type, which occurs in the absence of an underlying disease, and the secondary type, which is associated with a causative systemic disease. Among a number of potential causative antigens for MN that have been identified, phospholipase A2 receptor (PLA_2_R) and thrombospondin-type-1-domain-containing-7A (THSD7A), expressed on podocytes, are representatives for patients with idiopathic MN, and autoantibodies of the immunoglobulin (Ig)G4 subclass are dominant in the glomerular immune deposits of such patients.^[1,2]^

Although the precise disease pathogenesis remains to be clarified, predominant Th2 immune responses are supposed to play important roles in both MCD and MN.^[3]^ From the therapeutic point of view, efficacy and safety of rituximab, which targets B cells, is now widely recognized in both the diseases.^[4–6]^ However, whether some overlap exists between the 2 diseases is still unclear, and to the best of our knowledge, there is only 1 case reported that was initially diagnosed as MCD but subsequently developed into MN.^[7]^

Here, we have reported a case in which the first renal biopsy diagnosed MCD, but the repeat renal biopsy, which was performed approximately 4 years later owing to the relapse of proteinuria, demonstrated the development of THSD7A-associated MN during long-term steroid treatment.

## 2. Case presentation

**Figure 1.**
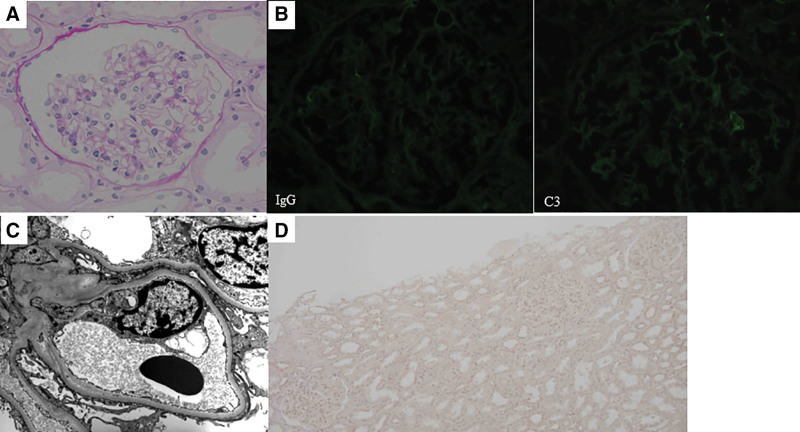
Histological features of the first renal biopsy. (A) Light microscopy image showing a glomerulus without proliferative changes and thickening of the glomerular capillary walls (periodic acid-Schiff stain). (B) Negative immunofluorescence staining for immunoglobulin G and complement C3. (C) Electron microscopy image showing effacement of podocyte foot processes. No electron-dense deposit was observed. (D) Immunoperoxidase staining for thrombospondin-type-1-domain-containing-7A was negative at this time.

A previously healthy Japanese man in his 50s developed anasarca, and was referred to our hospital 4 years ago. Massive proteinuria along with hypoproteinemia, which led to the diagnosis of nephrotic syndrome, was noted, as shown in Table 1. Despite low IgG level and high IgE level, dysproteinemia was not detected. Renal biopsy was performed, and light microscopy revealed glomeruli with apparently normal appearance (Fig. 1A). Immunofluorescence staining for Igs and complements was all negative (Fig. 1B), and no electron-dense deposit was observed by electron microscopy (Fig. 1C). He was diagnosed with MCD and was treated with steroids, as recommended by the guideline in Japan.^[8]^

**Table 1:** Laboratory data of the patient.

	At initial visit to our hospital	At repeat biopsy
Urinalysis		
Red blood cell	10–19/HPF	10–19/HPF
Proteinuria	11.10 g/g Cre	1.62g/g Cre
White blood cell	1–4/HPF	1–4/HPF
Complete blood count		
White blood cell	5580/µL	7740/µL
Eosinophil	212/µL	248/µL
Hemoglobin	13.3 g/dL	14.6 g/dL
Platelet	237,000/µL	237,000/µL
Biochemistry		
Creatinine	1.51 mg/dL	0.54 mg/dL
Blood urea nitrogen	30.6 mg/dL	9.1 mg/dL
Total protein	3.7 g/dL	5.6 g/dL
Albumin	0.8 g/dL	3.2 g/dL
Serology		
IgG/A/M	529/264/73 mg/dL	611/128/26 mg/dL
Complement C3/C4	108/49 mg/dL	144/36 mg/dL
IgE (RIST)	1030 IU/mL	199 IU/mL
Antinuclear antibody	40	<40
dsDNA-IgG	N.T.	<10 IU/mL
MPO-ANCA	<1.0 U/mL	N.T.
PR3-ANCA	<1.0 U/mL	N.T.
C-reactive protein	0.91 mg/dL	<0.02 mg/dL
HBs antigen	Negative	Negative
HCV antibody	Negative	Negative

ANCA = anti-neutrophil cytoplasmic antibody, dsDNA = double-stranded DNA, HBs = hepatitis B surface, HCV = hepatitis C virus, HPF = high-power field, Ig = immunoglobulin, MPO = myeloperoxidase, N.T. = not tested, PR3 = proteinase 3.

He achieved complete remission of nephrotic syndrome and maintained a stable condition under long-term steroid treatment. However, proteinuria (approximately 1 g/g Cr) recurred approximately 3 years after the onset of nephrotic syndrome. Although the dose of steroids was increased from prednisone at 7.5 mg daily to 25 mg daily, proteinuria aggravated further. Laboratory results at this point are summarized in Table 1. Although hypoproteinemia was noted, his serum creatinine level was normal. Dysmorphic red blood cells were observed in the urinary sediment, although nephritic casts were absent. He complained of peripheral edema, although the rest of the physical findings were unremarkable, and his vital signs were normal.

**Figure 2.**
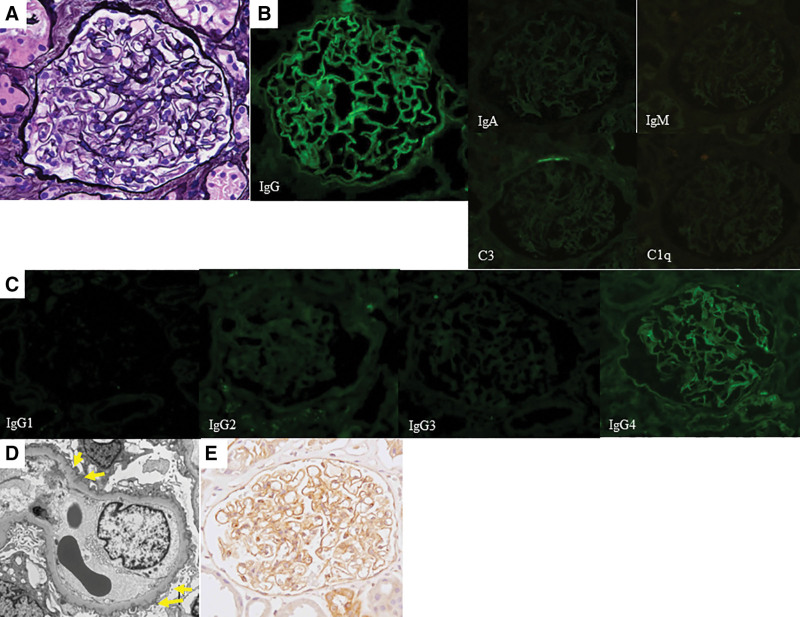
Histological features of the repeat renal biopsy. (A) Light microscopy image showing a glomerulus with almost normal appearance (periodic acid-methenamine-silver stain). (B) Immunofluorescence staining showing the deposition of immunoglobulin (Ig)G along the glomerular capillary walls. The deposition of IgA, IgM, and complements C3 and C1q was negative. (C) Dominant deposition of IgG4 is shown by immunofluorescence staining of the IgG subclasses. (D) Electron microscopy demonstrated small amounts of subepithelial electron-dense deposits (yellow arrows) and podocyte foot process effacement. Spike formation of the glomerular basement membrane was unclear. (E) Immunoperoxidase staining for thrombospondin-type-1-domain-containing-7A was strongly positive on the glomerular capillary walls.

Since the patient presented with atypical clinical findings of MCD, such as, steroid-resistant proteinuria accompanied by glomerular hematuria, repeat renal biopsy was performed approximately 4 years after the first. Light microscopy sections contained 16 glomeruli, of which one was obsolescent, but neither proliferative changes nor thickening of the glomerular capillary walls was seen in the remaining glomeruli (Fig. 2A). Immunofluorescence staining showed deposition of IgG along the glomerular capillary walls, although that of IgA, IgM, and complements C3 and C1q was negative (Fig. 2B). Immunofluorescence staining for IgG subclasses showed the dominant deposition of IgG4 (Fig. 2C). Although electron microscopy showed small amounts of subepithelial electron-dense deposits, spike formation of the glomerular basement membrane was unclear (Fig. 2D). Immunostaining for THSD7A was positive on the glomerular capillary walls (Fig. 2E), whereas that for PLA_2_R or neural epidermal growth factor-like 1 protein was negative (data not shown). Serum anti-PLA_2_R, measured by the enzyme-linked immunosorbent assay, was negative (2.04 RU/mL; positive cutoff value, 20 RU/mL).

Detailed examination, including imaging tests, was repeatedly performed, but underlying diseases, such as malignancies, were not found, and a diagnosis of THSD7A-associated idiopathic MN (stage I) was made based on the second renal biopsy. He received further increased dose of steroids [intravenous half-dose methylprednisolone pulse therapy (500 mg daily for 3 days) followed by oral prednisone at 30 mg per day]. His urinary protein level gradually decreased thereafter, and he maintained clinical improvement during the 1-year follow-up period (proteinuria level of approximately 0.5 g/g Cr with normal serum creatinine level).

## 3. Discussion

We reported a case in which histological transition from MCD to MN occurred during long-term steroid treatment. To the best of our knowledge, there had only been one such case reported to date, in which first and second renal biopsy yielded the diagnosis of MCD and idiopathic MN, respectively, but third renal biopsy resulted in the final diagnosis of lupus nephritis.^[7]^ In the reported case, causative antigen for idiopathic MN was not identified, and complement C1q deposition, which suggests the possibility of secondary MN, was observed. On the other hand, in our patient, C1q deposition was negative, and dominant IgG4 deposition by immunofluorescence IgG subclass staining was observed. Furthermore, enhanced granular expression of THSD7A on the glomerular capillary walls prompted the final diagnosis of THSD7A-associated idiopathic MN.

THSD7A is a second antigenic target for idiopathic MN; it was identified in 2014, followed by the first discovery of PLA_2_R.^[2]^ The prevalence of THSD7A-associated MN in idiopathic MN is reportedly 3 to 9% in Japan and is supposed to be higher than that in western countries.^[9,10]^ Moreover, THSD7A-associated MN is reported to often be associated with malignancies.^[11]^ Some studies have reported THSD7A-associated MN to be related to allergic diseases, especially asthma, or eosinophilia.^[12,13]^ In our patient, detailed and repeated examination did not detect malignancies or allergic diseases. The patient’s eosinophil count was normal when THSD7A-associated MN was diagnosed, although assessment was difficult due to the administration of steroids. Nonetheless, careful and prolonged follow-up, to monitor the development of malignancy, is warranted, since the onset of MN is known to sometimes precede the development of malignancy over the years. In addition, it is reported that the etiology of MN could change from idiopathic to malignancy-associated MN during the clinical course.^[14]^ Moreover, previous diagnosis of MCD and MN does not necessarily preclude the possibility of subsequent development of systemic lupus erythematosus, as reported earlier.^[7]^

Predominant Th2 immune responses are supposed to play important roles in both MCD and MN.^[3]^ Our patient presented elevated serum IgE level when he first developed MCD, although we did not evaluate the patient’s other immune conditions, such as Th1/Th2 cytokines or thymus and activation-regulated chemokine. Repeat renal biopsy is now seldom performed in patients with both MCD and MN; however, the present case suggested that susceptible individuals could develop both MCD and MN, depending on as-yet-unknown pathogenetic mechanisms, and that histological transition from one to another could occur. Further accumulation and analyses of similar cases would be required to clarify this point. Moreover, the importance of repeat renal biopsy, which is crucial for definitive diagnosis, should be realized especially when unexpected clinical courses occur.

The positive rate of serum anti-THSD7A antibodies has been reported to be comparable to that observed by glomerular THSD7A immunostaining.^[15]^ Although immunostaining for THSD7A, using the first renal biopsy tissue, was negative (Fig. 1D), we could not evaluate the patient’s serum anti-THSD7A antibodies throughout the clinical course due to the lack of preserved serum samples, which is a limitation of this report. Nevertheless, the present case showed histological transition from MCD to THSD7A-associated MN during long-term steroid treatment, suggesting that when unexpected clinical courses occur, renal biopsy should be performed repeatedly. Further, whether there exists some commonality between MCD and MN should be investigated in a future study.

## Acknowledgments

We thank our colleague, Ms. Sachiko Iwama, for her excellent technical assistance, Ms. Yukari Kawamura, for expert secretarial assistance, and Editage (www.editage.jp), for English-editing of the manuscript.

## Author contributions

**Conceptualization:** Takahiro Uchida, Takashi Oda.

**Data curation:** Aki Kojima, Kentaro Sugisaki, Minami Koizumi, Ken Aoki, Mitsuya Mukae.

**Supervision:** Takahiro Uchida, Takashi Oda.

**Writing – original draft:** Aki Kojima, Takahiro Uchida.

**Writing – review & editing:** Muneharu Yamada, Takashi Oda.
